# Supplementary material for: Assessing the Relationship of Different Levels of Pain to the Health Status of Long-Term Breast Cancer Survivors: A Cross-Sectional Study
Source: Life (Basel). 2025 Jan 25;15(2):177. doi: 10.3390/life15020177 (PMC11856788; doi:10.3390/life15020177)
Supplement: Supplementary file 1 [file life-15-00177-s001.zip › Supplementary Figure S1. Flow Diagram.pdf]

Supplementary Figure S1. Flow diagram for study participants

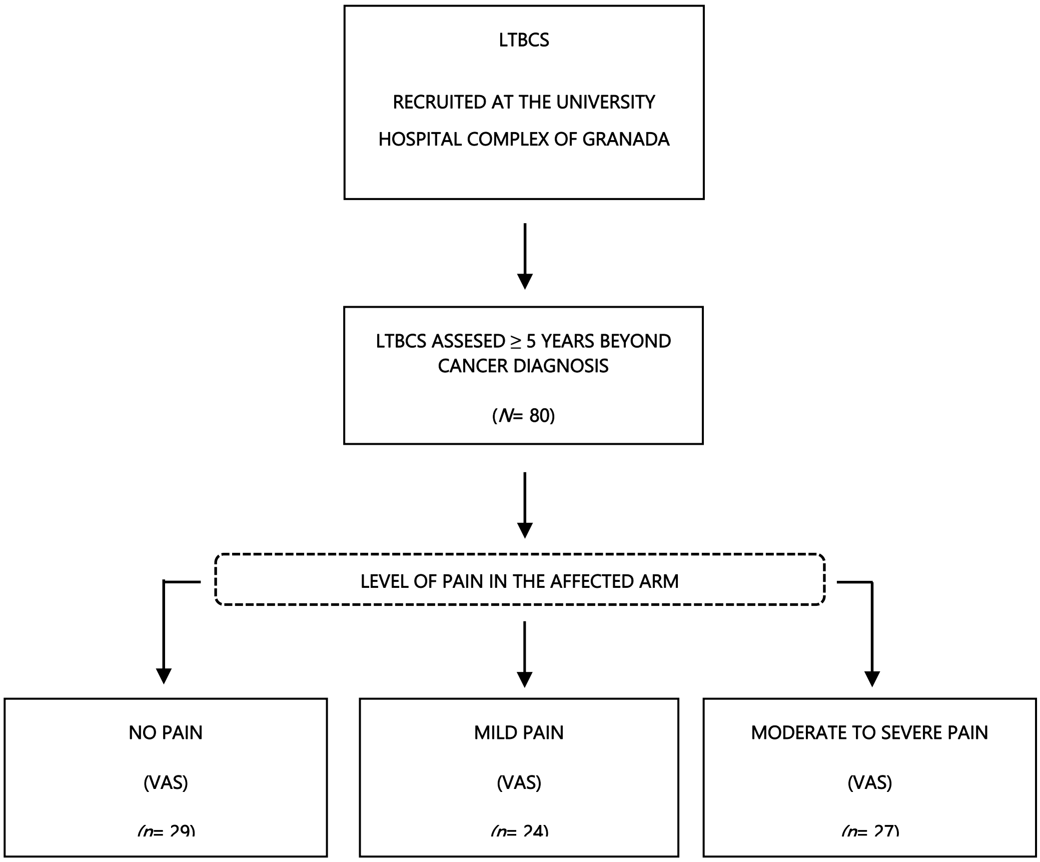

Abbreviations: *LTBCS* Long-term breast cancer survivors, *VAS* Visual Analogue Scale, *N*/*n* simple size
