# Supplementary material for: Assessing the Relationship of Different Levels of Pain to the Health Status of Long-Term Breast Cancer Survivors: A Cross-Sectional Study
Source: Life (Basel). 2025 Jan 25;15(2):177. doi: 10.3390/life15020177 (PMC11856788; doi:10.3390/life15020177)
Supplement: Supplementary file 1 [file life-15-00177-s001.zip › Supplementary Table S1. Fitness condition.pdf]

Supplementary Table S1. Fitness condition of LTBCS according to the level of pain in the affected arm

VARIABLES	LTBCS LEVEL OF PAIN IN THE AFFECTED ARM								
	NO PAIN (NP)	MILD PAIN (MP)	MODERATE TO SEVERE PAIN (MTSP)						
	0 – 0.99 (VAS)	1 – 3.99 (VAS)	4 – 10 (VAS)	*P-values*	COHEN'S *d*	*P-values*	COHEN'S *d*	*P-values*	COHEN'S *d*
	(*n*= 29)	(*n*= 24)	(*n*= 27)	NP vs MP	NP vs MP	NP vs MTSP	NP vs MTSP	MP vs MTSP	MP vs MTSP
IFIS, mean ± SD, median ; IQR, and (95% CI)									
General physical fitness	3.90 ± 0.91<br>4.00 ; 2.00<br>(3.58 – 4.23)	2.90 ± 0.96<br>3.00 ; 0.75<br>(2.44 – 3.35)	2.81 ± 0.68<br>3.00 ; 1.00<br>(2.54 – 3.08)	<0.01**	1.0	<0.01**	1.35	0.67	0.10
Cardiorespiratory fitness	3.24 ± 1.17<br>3.00 ; 1.05<br>(2.82 – 3.65)	2.40 ± 0.99<br>2.00 ; 1.00<br>(1.93 – 2.86)	2.81 ± 0.96<br>3.00 ; 1.00<br>(2.43 – 3.19)	0.01*	0.88	0.02*	0.45	0.17	0.42
Muscular strength	3.33 ± 1.08<br>3.00 ; 1.00<br>(2.95 – 3.71)	2.70 ± 0.86<br>3.00 ; 1.00<br>(2.29 – 3.10)	2.44 ± 0.84<br>2.00 ; 1.00<br>(2.10 – 2.77)	0.02*	0.64	<0.01**	0.91	0.27	0.30
Speed / Agility	3.36 ± 0.99<br>3.00 ; 1.00<br>(3.01 – 3.71)	2.50 ± 0.82<br>2.50 ; 1.00<br>(2.11 – 2.88)	2.77 ± 0.84<br>3.00 ; 1.00<br>(2.44 – 3.11)	0.01**	0.94	<0.01**	0.64	0.37	0.32
Flexibility	3.42 ± 0.90<br>4.00 ; 1.00<br>(3.10 – 3.74)	2.40 ± 0.88<br>2.00 ; 1.00<br>(1.98 – 2.81)	2.55 ± 1.01<br>3.00 ; 1.00<br>(2.15 – 2.95)	<0.01**	1.14	<0.01**	0.90	0.55	0.15

Abbreviations: *LTBCS* Long-term Breast Cancer Survivors, *VAS* Visual Analog Scale, *IFIS* International Fitness Scale, *NP* No Pain, *MP* Mild Pain, *MTSP* Moderate to Severe Pain, *CI* Confidence interval, *n* Sample size, *SD* Standard deviation, *IQR* Inter-Quartile Range

P values for between-group differences were calculated using the t test (Mann-Whitney U test)

Between-group effect sizes were calculated using Cohen's d

*P < 0.05

**P < 0.01
